# Audit on monitoring physical health of patients on mood stabilisers following NICE guidelines

**DOI:** 10.1192/bjo.2021.311

**Published:** 2021-06-18

**Authors:** Sathyan Soundararajan, Asha Dhandapani, Claire Jones

**Affiliations:** BCUHB

## Abstract

**Aims:**

The aim is to find out if the physical health monitoring is adhered to in accordance with NICE guidelines in individuals with Intellectual disability who are on mood stabilisers and known to LD services.

**Method:**

We sought to explore if the physical health monitoring for prescribing mood stabilisers in a sample of people with ID was consistent with good practice guidelines.

We collected the data by reviewing the clinical records of individuals with LD who were under the care of mental health services in the CLDT- Wrexham and prescribed a mood stabiliser drug. We also contacted the patient's carers who came to outpatients and by calling the GP surgery and enquiring about the details. We also assessed the Welsh clinical portal in order to assess the blood tests.

Data were collected by trainee doctors in Psychiatry. This was a retrospective audit, looking at data from Learning Disability psychiatry caseload. We identified about 16 patients on mood stabilisers.

**Result:**

Physical health monitoring for prescribing mood stabilisers was almost consistent with good practice guidelines. This has shown that the majority of the monitoring has complied. There are few lacunae, such as Thyroid function not being monitored every 6 months for patients on Lithium, Serum Carbamazepine levels not being monitored as per guidelines with 1 patient not having blood done at all whilst on Carbamazepine. Moreover, the details are not readily available for the Consultant/ team when needed, thus making it very tedious for them to search/ contact the GP, etc.

**Conclusion:**

Medications such as mood stabilisers can increase the risk further if the patient's physical health is not monitored regularly. This can lead to compromised quality of life for the patient and in some cases increased morbidity. Hence we have come up with a proforma that can be attached to patient case notes. This will serve as a record for us and prompt for physical monitoring. We will keep a database online with reminders set. This is to ensure a continuity of care for the patients.

